# Identifying Learning Preferences and Strategies in Health Data Science Courses: Systematic Review

**DOI:** 10.2196/50667

**Published:** 2024-08-12

**Authors:** Narjes Rohani, Stephen Sowa, Areti Manataki

**Affiliations:** 1 Usher Institute University of Edinburgh Edinburgh United Kingdom; 2 Moray House School of Education and Sport University of Edinburgh Edinburgh United Kingdom; 3 School of Computer Science University of St Andrews St Andrews United Kingdom

**Keywords:** health data science, bioinformatics, learning approach, learning preference, learning tactic, learning strategy, interdisciplinary, systematic review, medical education

## Abstract

**Background:**

Learning and teaching interdisciplinary health data science (HDS) is highly challenging, and despite the growing interest in HDS education, little is known about the learning experiences and preferences of HDS students.

**Objective:**

We conducted a systematic review to identify learning preferences and strategies in the HDS discipline.

**Methods:**

We searched 10 bibliographic databases (PubMed, ACM Digital Library, Web of Science, Cochrane Library, Wiley Online Library, ScienceDirect, SpringerLink, EBSCOhost, ERIC, and IEEE Xplore) from the date of inception until June 2023. We followed the PRISMA (Preferred Reporting Items for Systematic Reviews and Meta-Analyses) guidelines and included primary studies written in English that investigated the learning preferences or strategies of students in HDS-related disciplines, such as bioinformatics, at any academic level. Risk of bias was independently assessed by 2 screeners using the Mixed Methods Appraisal Tool, and we used narrative data synthesis to present the study results.

**Results:**

After abstract screening and full-text reviewing of the 849 papers retrieved from the databases, 8 (0.9%) studies, published between 2009 and 2021, were selected for narrative synthesis. The majority of these papers (7/8, 88%) investigated learning preferences, while only 1 (12%) paper studied learning strategies in HDS courses. The systematic review revealed that most HDS learners prefer visual presentations as their primary learning input. In terms of learning process and organization, they mostly tend to follow logical, linear, and sequential steps. Moreover, they focus more on abstract information, rather than detailed and concrete information. Regarding collaboration, HDS students sometimes prefer teamwork, and sometimes they prefer to work alone.

**Conclusions:**

The studies’ quality, assessed using the Mixed Methods Appraisal Tool, ranged between 73% and 100%, indicating excellent quality overall. However, the number of studies in this area is small, and the results of all studies are based on self-reported data. Therefore, more research needs to be conducted to provide insight into HDS education. We provide some suggestions, such as using learning analytics and educational data mining methods, for conducting future research to address gaps in the literature. We also discuss implications for HDS educators, and we make recommendations for HDS course design; for example, we recommend including visual materials, such as diagrams and videos, and offering step-by-step instructions for students.

## Introduction

### Background

In the era of artificial intelligence, big data, and digitalization of health care, there is a growing demand for educating specialists in analyzing health data [1-3]. The integration of IT into health care has undergone significant evolution in recent decades that has led to a change in the definition of health informatics. The current definition of health informatics encompasses the interdisciplinary study of designing, developing, adopting, and applying IT-based innovations in health care service delivery, management, and planning. By contrast, health care data analytics, a nascent subfield within health informatics, specifically addresses methods and techniques for analyzing, integrating, and interpreting health care data. Health data analytics, or health data science (HDS), as it can also be understood, involves data manipulation, mining, and statistical analysis to gain valuable insights from health, medical, or biological data. In other words, while health informatics encompasses noncomputational aspects, such as system development and maintenance, health data analytics or HDS concentrates only on using computational tools and methods for analyzing data [4].

However, given the novel and interdisciplinary nature of HDS, learning and teaching HDS is highly challenging [1,5,6]. Students and teachers are often faced with a lack of common language and prior knowledge in health or computational sciences, thus making it hard to learn and teach HDS concepts effectively [1,7]. In postgraduate study, in particular, students who enroll in HDS courses have diverse academic backgrounds, including computational and medical backgrounds (but rarely a combination of the two); therefore, traditional learning and teaching approaches in biology, medicine, or computer sciences may not be effective for HDS training [1,8].

Shedding light on HDS students’ learning preferences and strategies is particularly important in this context and can help address some of these challenges [7,9-12]. There is heterogeneous literature around the definitions of *learning strategy*, *learning tactic*, *learning approach*, *learning style*, and other related terms [10,13,14,15]. In this paper, we view learning strategy as the approach that students use to manage their learning processes.

Similar to recent studies [16-19], we also understand learning preference as the perceived tendency of learners regarding the presentation of learning materials, types of learning activities, and the organization of their learning process, while learning strategy or learning approach is the actual way in which students manage their learning process [19].

We also recognize that the learning preferences that students exhibit within the HDS field inform the strategies they use to support their learning [20,21]. We decided to focus on learning preferences and strategies from the aforementioned perspectives because these field-specific preferences and strategies can offer insights into HDS education, which are useful for personalized learning [17,22-24].

Given the aforementioned definition of learning preference, research studies about learning styles in HDS-related fields touch upon HDS-specific learning preferences and can thus be used to identify students’ tendencies in the field regarding information presentation, learning activities, and learning organization. However, it should be mentioned that the term *learning style* has been consistently misinterpreted [18] and defined variably across numerous studies in the literature [18,25]. In recent years, several research studies [26,27] have criticized the claim that each individual student has a dominant learning style, which is a stable neurological, psychological, or innate learning preference. Nonetheless, these and other studies [10,12,16,18,26,27] have also acknowledged that students in each field of study, specific to the nature of the discipline, might exhibit some preferences regarding course materials and activities and the way in which they approach these materials and activities [10,12,16,18,26]. As mentioned in a previous study [26], while the concept of stable learning styles for students is considered a myth, there are preferences that students exhibit within each field that informs the strategies they use to support their learning, which can in turn support personalized learning [10,11,16,18,20,26,28-30].

Given this context, gaining knowledge about learners’ preferences and strategies in HDS courses can help course designers create optimized courses or redesign existing courses [10,31,32], creating a positive impact on student interest, engagement, and performance [16,32]. In addition, informing teachers about students’ learning preferences and strategies in HDS courses can assist them not only in selecting appropriate teaching methods but also in providing personalized feedback to students [10,30,33,34].

Although several systematic reviews have been conducted to investigate the learning preferences of nurses [35,36] and physiotherapists [37], none of them are related to interdisciplinary programs in the realm of HDS. To fill this gap, and following the PRISMA (Preferred Reporting Items for Systematic Reviews and Meta-Analyses) guidelines [38], we conducted a systematic review to present the current state of knowledge on learning strategies and preferences in HDS.

There are important aspects of learning strategies and preferences that are of interest in this systematic review because they are useful for implementing personalized learning in the HDS field [11,21]. The types of multimedia resources in a course are important because they significantly influence engagement, understanding, and the overall learning experience of students [39,40]. Each discipline has its unique nature [10,26], and presenting concepts in an effective way that is aligned with students’ preferences in the discipline can improve students’ satisfaction [41]. Therefore, insight into preference regarding the types of multimedia resources used for information delivery can enhance course design and student satisfaction.

Collaborative learning is one of the popular strategies in education, but it is not always easy to implement it successfully because engaging all students in teamwork is challenging [42-44]. Therefore, understanding students’ collaboration preferences in HDS can facilitate the integration of both peer learning and independent study within a course to improve collaborative skills, support diverse perspectives, and help students to develop self-directed learning skills [42-44].

In addition, understanding whether HDS students prefer a global or sequential approach when studying topics can inform both teachers and students about effective learning strategies to enhance the student educational journey; for example, course designers can arrange topics in more effective sequences that align better with students’ preferences, thereby improving the overall learning experience [45].

Moreover, understanding the preferred focus granularity of students, such as their inclination toward details or abstract concepts, assists in prioritizing topics for teaching and determining effective teaching strategies [46,47]; for example, identifying whether HDS students prefer applied topics or theoretical aspects helps educators decide the level of details to include in the course materials [47]. These are all important topics related to learning strategies and preferences, which are worth shedding light on in the context of HDS education.

### Research Questions

Therefore, this systematic review focuses on the following research questions (RQs), which were selected based on available literature and their potential benefits for personalized learning [20,21]:

- RQ1: What types of information presentation do students prefer in HDS?
- RQ2: Do students prefer team-based learning over independent learning in HDS?
- RQ3: How do students organize their learning process (global vs sequential) in HDS?
- RQ4: Do students in HDS prefer abstract concepts over factual concepts?

Our goal with this systematic review is not only to present and analyze research findings on learning strategies and preferences in HDS but also to discuss their implications for future course design in HDS. This way, we can help HDS educators make informed decisions about teaching methods and assist them with developing effective courses. To the best of our knowledge, this is the first systematic review that discusses learning strategies and preferences in HDS-related disciplines. The contributions of this study are as follows:

- It consolidates the heterogeneous knowledge available in the literature and presents it in 4 categories, that is, information presentation (RQ1), collaboration preference (RQ2), organization strategy (RQ3), and focus granularity (RQ4).
- It provides suggestions to assist course designers and teachers in delivering more effective HDS-related courses.
- It provides suggestions for future research in HDS education, which can help researchers conduct better informed investigations in this area.

## Methods

### Overview

This systematic review was conducted to understand what learning strategies and preferences are used by students in HDS-related fields. To this end, we followed all steps outlined in the PRISMA guidelines [38] except for the meta-analysis step because, given the diversity of the included papers, the narrative data synthesis approach [48] was deemed more appropriate for combining the findings from the different studies. Therefore, we used narrative data synthesis to report our findings. The PRISMA checklists for abstracts and articles are available in Tables S1 and S2 in Multimedia Appendix 1, respectively. We also used the Mixed Methods Appraisal Tool (MMAT) [49] to assess the quality of the included articles. The MMAT allows the assessment of the quality of studies with different methodological designs, such as quantitative, qualitative, and mixed designs. The protocol used in this study is available in Multimedia Appendix 2 [38,48-51], and the PICO (Population, Intervention, Comparison, and Outcomes) components of the review question are presented in Table S1 in Multimedia Appendix 2.

### Types of Studies and Participants

In this systematic review, we considered various types of primary studies, including both quantitative and qualitative journal or conference papers, all of which focused on exploring learning preferences or strategies in HDS-related courses. We did not apply any restrictions regarding participants’ academic degrees; therefore, all high school, undergraduate, and postgraduate students as well as nontraditional learners (eg, health care professionals) were included.

### Study Eligibility

This systematic review focuses on courses and programs falling within the scope of HDS (using data analytics methods to analyze biological, medical, and health data) [4,7]. Studies focusing on non–data analytics aspects of health informatics were not considered in this systematic review.

The inclusion criteria are presented in Textbox 1 (for more study eligibility details, refer to Table S2 in Multimedia Appendix 2).

**Criteria used to select the studies.**

**Inclusion criteria**

- Language of publication: English
- Year of publication: no restriction applied regarding the year of publication
- Participants: students in fields highly relevant to health data science (HDS; using computational methods for medical, biological, or health data analysis), such as bioinformatics, biostatistics, computational biology, neuroinformatics, biomedical science, precision medicine, HDS, and HDS courses
- Participants’ academic level: high school, undergraduate, and postgraduate students in any relevant course; nontraditional learners, such as health care professionals, also included
- Type of publication: conference and journal papers; primary research articles
- Subject: papers discussing learning preferences, strategies, tactics practices, or styles of the aforementioned learners
- Analysis type: both quantitative and qualitative methods included

### Study Identification

The literature search was carried out on June 15, 2023. The PubMed, ACM Digital Library, Web of Science, Cochrane Library, Wiley Online Library, ScienceDirect, SpringerLink, EBSCOhost, ERIC, and IEEE Xplore databases were searched independently. We supplemented the literature search by using Google Scholar manually to find potentially missed articles. Given the interdisciplinary nature of HDS, these databases were selected to cover literature across computer science, education, and medicine. We used a combination of terms to identify papers about students’ learning preferences and strategies in a variety of courses and programs related to HDS. The keywords used for searching the literature are presented in Table S3 in Multimedia Appendix 2, and the queries used for each database are presented in Table S4 in Multimedia Appendix 2.

### Study Selection

The title, abstract, and full-text screening were carried out independently by 2 reviewers: NR, who has an academic background in HDS; and SS, who has a background in education. They screened the titles and abstracts of all extracted articles, followed by a full-text review of eligible studies (Cohen κ agreement index=0.95). In cases of disagreement, a third screener, AM, was involved to resolve conflicts. The screening questions are presented in Table S5 in Multimedia Appendix 2.

### Data Extraction

Both NR and SS used a standardized Microsoft Word form for extracting and documenting data (for details, refer to Multimedia Appendix 2). The data they extracted included the following categories: publication characteristics (this included details such as the publication title, journal or conference, authors, and publication year); methodological features (the reviewers recorded various methodological aspects, such as the participants’ field and course name, the number of participants, the method of analysis used, the type of input data used, the students’ degree level, the study subject, and any learning inventory used); and learning preference or learning strategy (information regarding reported learning preferences or strategies was collected, along with the corresponding percentage of students exhibiting each learning preference or strategy).

After the initial extraction, both reviewers cross-checked the extracted data to ensure accuracy. In addition, both reviewers assessed the quality of the included articles independently by using the MMAT [49]. Finally, any discrepancies or inconsistencies were independently resolved by the third reviewer, AM.

## Results

### Search Results

**Figure 1:**
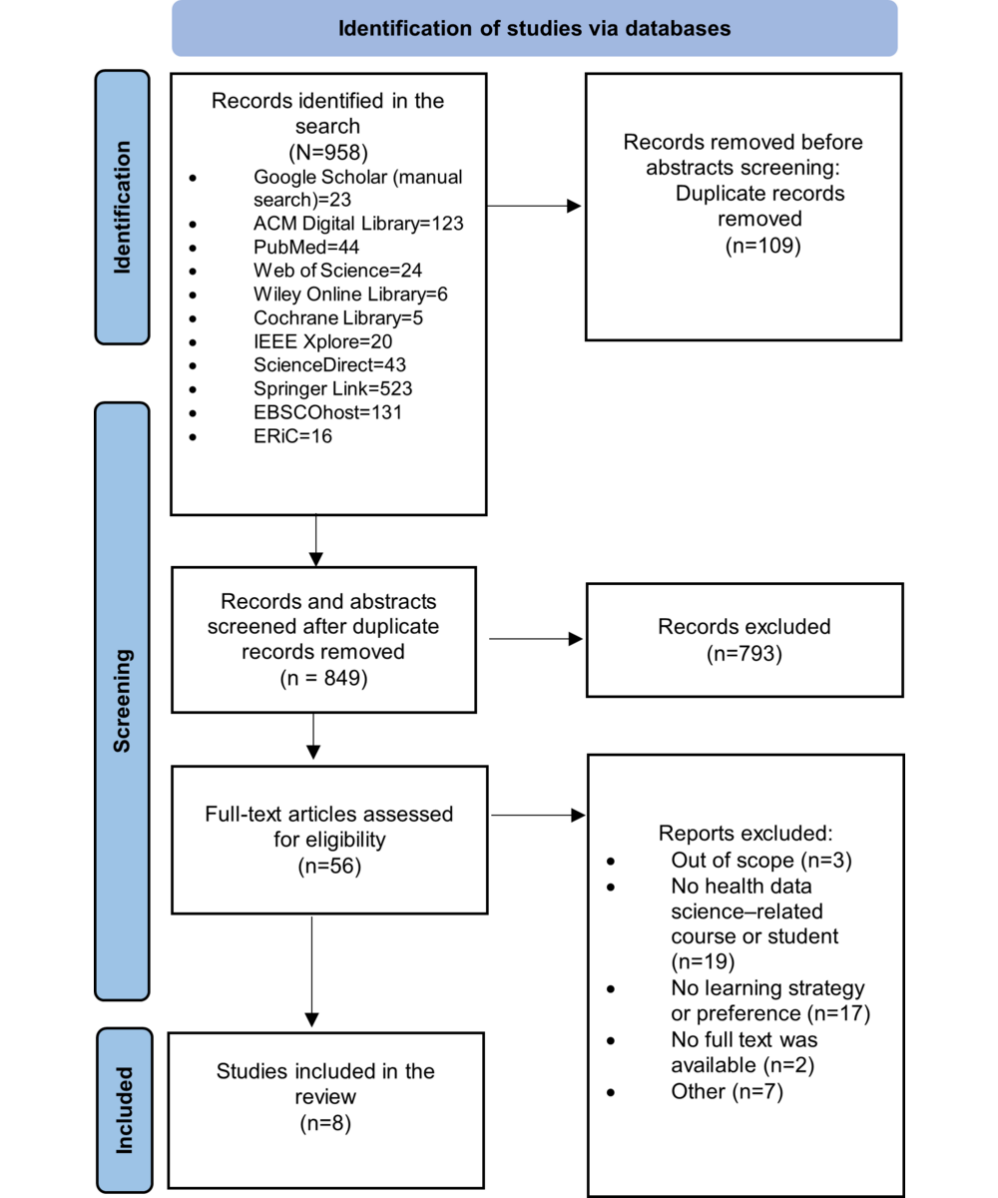
PRISMA (Preferred Reporting Items for Systematic Reviews and Meta-Analyses) flowchart of the study selection process. Full texts could not be found for 2 (4%) of the 56 papers considered for full-text review after abstract screening.

The literature search resulted in 958 articles, which were reduced to 849 (88.6%) studies after removing 109 (11.4%) duplicates (for details, refer to Figure 1). Of these 849 articles, after full-text review, 8 (0.9%) studies that were published between 2005 and 2021 were included in the synthesis. The reasons for excluding papers during full-text screening are presented in Table S6 in Multimedia Appendix 2.

### Characteristics of the Included Studies

As shown in Table 1, most of the articles (7/8, 88%) were published between 2017 and 2021. Of the 8 studies, 2 (25%) were conducted in the United States; 2 (25%) in Malaysia (2/8, 25%); and 1 (12%) each in Denmark, India, Sweden, and Israel. Of the 8 studies, 3 (38%) [45,46,52] focused on undergraduate students, and 3 (38%) focused on postgraduate students [53-55], while high school learners were investigated by 1 (12%) study [56], and health care professionals were the focus of 1 (12%) study [41].

**Table 1:** Summary of the included studies.

Study, year; country	Sample size, n	Participants’ field	Course	Course delivery type	Participants’ academic level	Study subject	Learning inventory	Results
Holtzclaw et al [45], 2017; United States	28	Genetics	Bioinformatics	Face-to-face with web-based materials	Undergraduate student	Learning style	FSILS^a^	Procession: active=54%^b^, reflective=46%; input: visual=82%, verbal=18%; perception: sensing=67%, intuitive=33%; understanding: sequential=79%, global=21%
Micheel et al [41], 2017; United States	751	Oncology	Precision medicine	Web based	HCP^c^	Learning style	Custom survey with 1 question	Multimodal (80%): watching, listening, and reading=39%, watching and reading=19%, listening and reading=12%, watching and listening=10%; unimodal (20%): reading=15%, watching=3%, listening=2%
Nielsen and Kreiner [53], 2017; Denmark	57	Public health	Advanced statistics	Face-to-face	Postgraduate student	Learning style	D-SA-LSI^d^ and qualitative analysis	Function: executive=5.42^e^ (strong), legislative=4.59 (strong), judicial=4.41 (medium); form: democratic=4.62 (strong), anarchic=4.34 (medium), monarchic=3.68 (medium), hierarchic=4.12 (medium), oligarchic=2.65 (weak); learning: conservative=4.54 (strong), progressive=4.83 (strong); level: global=3.97 (medium), local=3.59 (medium); scope: external=5.43 (strong), internal=3.53 (medium)
Diwakar et al [54], 2018; India	84	Biotechnology, microbiology, and bioinformatics	Bioinformatics and biotechnology	Web based	Postgraduate student	Learning style	Kolb learning style inventory	Assimilators=60%, divergers=20%, convergers=16%, accommodators=4%
Sani Ibrahim [46], 2020; Malaysia and Nigeria	2 data sets were used: procession data set=95, perception data set=2168	Bioinformatics	Genomics technology	Web based	Undergraduate student	Learning style	FSILS and data mining	Procession: active=70%, reflective=30%; perception: intuitive=94%, sensing=6%
Abrahamsson and Dávila Lopez [55], 2021; Sweden	65	Bioinformatics	Bioinformatics	Web based and face-to-face	Postgraduate student	Learning style	Custom survey and qualitative analysis	Lecture format: real-time Zoom sessions=64%, offline as a video=27%, offline as reading=9%; synchronize work preference: alone=50%, alone and then in group=12%, same group=19%, different group=19%
Gelbart et al [56], 2009; Israel	4	Biology	Bioinformatics	Face-to-face with web-based materials	High school student	Learning strategy or approach	Custom survey and qualitative analysis	1 pair research oriented and 1 pair task oriented
Li and Abdul Rahman [52], 2018; Malaysia	46	Bioinformatics	Genomics technology	Web based	Undergraduate student	Learning style	FSILS and data mining	Procession: active=55%, reflective=24%, neutral=21%; input: visual=66%, verbal=18%, neutral=16%; perception: sensing=31%, intuitive=48%, neutral=21%; understanding: sequential=62%, global=12%, neutral=26%

^a^FSILS: Felder and Soloman Index of Learning Styles.

^b^The numbers denoted by a percentage sign in the Results column represent the percentage of learners who have declared the corresponding learning preference among all learners.

^c^HCP: health care professional.

^d^D-SA-LSI: Danish Self-Assessment Learning Styles Inventory.

^e^The scores indicate the strength of students’ inclination toward the corresponding preference and were calculated based on the D-SA-LSI (range 0-7).

Of the 8 included studies, 6 (75%) [45,46,52,54-56] explored the learning strategies and preferences of bioinformatics students or courses, 1 (12%) investigated a precision medicine course [41], and 1 (12%) investigated an advanced statistics [53] course. It is worth noting that none of the included studies focused on courses specifically labeled as “health data science” courses.

Of the 8 included studies, 7 (88%) explored learning preferences, while 1 (12%) [56] analyzed students’ learning strategies. Slightly more than one-third of the studies (3/8, 38%) [41,55,56] used a custom survey to measure students’ learning preferences or strategies, while the rest (5/8, 62%) [45,46,52-54] used learning inventories, which are questionnaires that categorize students into different groups based on various learning dimensions (for a detailed description, refer to Multimedia Appendix 3 [57-63]). Of the 8 included studies, 3 (38%) [45,46,52] used the Felder and Soloman Index of Learning Styles (FSILS) [58,59], 1 (12%) [54] used the Kolb learning style inventory [57], and 1 (12%) [53] used the Danish Self-Assessment Learning Styles Inventory based on the theory propounded by Sternberg [60,64,65].

Regarding the analysis approach and data, most of the articles (6/8, 75%) performed only a qualitative analysis using a questionnaire and simple quantitative methods, such as statistical descriptive techniques applied to questionnaires (3/6, 50%) [53,55,56]. Three of them also supplemented their studies with a qualitative method. However, 2 (25%) of the 8 papers [46,52] used advanced data mining methods, such as k-means, and analyzed log data alongside self-reported data. Nevertheless, these studies [46,52] did not use log data to identify students’ learning preferences; instead, they relied on self-reported inventories to train their models. Furthermore, all included studies except that by Micheel et al [41] had sample sizes of <100 (average 65) participants. The characteristics of the included articles are illustrated using various visualizations in Figures S1 and S2 in Multimedia Appendix 2.

The studies’ quality, assessed using the MMAT, ranged between 73% and 100%, indicating excellent quality overall. None of the studies were excluded based on the MMAT score. Further details regarding the quality of the included articles and the MMAT checklists can be found in Multimedia Appendix 4 [41,45,46,52-56].

We used the narrative data synthesis approach [48] to combine the included studies to identify the learning preferences and strategies used in HDS. The studies were synthesized and narrated across different aspects, including information presentation preference (RQ1), collaboration preference (RQ2), preferred organization of learning process (RQ3), and preferred focus granularity (RQ4).

### Proxies Used for Synthesis

Due to the heterogeneity among the included studies in terms of the measurements used to determine learning preferences and strategies in HDS courses, we found it necessary to define specific proxies for each learning preference. These proxies help in making connections between the results presented in the different studies. Table 2 displays the proxies associated with each RQ in this systematic review. More information about the learning inventories discussed in the included studies is available in Multimedia Appendix 3.

**Table 2:** Proxies used to connect the included studies’ results to the research questions (RQs). The supporting evidence column provides available evidence in the literature about the association between the learning preference, style, or strategy and the proxies used.

Learning preference or strategy	Proxy	Source of the learning preference or strategy	Supporting evidence	RQ
Watching	Visual	Customized survey designed by Micheel et al [41]	Tendency toward watching lectures can be equivalent to a preference for visuals [41]	RQ1
Lecture	Visual	Customized survey designed by Abrahamsson and Dávila Lopez [55]	Tendency toward watching lectures can be equivalent to a preference for visuals [55]	RQ1
Assimilator	Visual	Kolb learning style inventory [57]	Assimilators are interested in learning through visual materials, such as videos and figures [57]	RQ1
Active	Teamwork	Felder and Soloman Index of Learning Styles [58,59]	Active students tend to work as a group and discuss learning materials with others [58,59]	RQ2
External	Teamwork	Danish Self-Assessment Learning Styles Inventory based on the theory propounded by Sternberg [60,65]	External students tend to work in a team and collaborate with others to solve problems [60,65]	RQ2
Internal	Independent work	Danish Self-Assessment Learning Styles Inventory based on the theory propounded by Sternberg [60,65]	Internal students prefer to work alone without communication with others [60,65]	RQ2
Reflective	Independent work	Felder and Soloman Index of Learning Styles [58,59]	Reflective learners are inclined to work alone or communicate with a close friend instead of a large group [58,59]	RQ2
Sequential	Sequential	Felder and Soloman Index of Learning Styles [58,59]	Sequential students have a linear learning process, which means they prefer to gain knowledge by following incremental and logical steps [58,59]	RQ3
Assimilator	Sequential	Kolb learning style inventory [57]	Assimilator students can organize the gained knowledge in a logical and clear format [57]	RQ3
Sensing	Factual information	Felder and Soloman Index of Learning Styles [58,59]	Sensing learners are interested in facts and concrete concepts, and they prefer exploring detailed information and intend to solve problems with standard approaches rather than innovative ones [58,59]	RQ4
Intuitive	Abstract information	Felder and Soloman Index of Learning Styles [58,59]	Intuitive learners are enthusiastic about abstract information, such as theories, and the deep meaning of learning materials [58,59]	RQ4
Global	Abstract information	Danish Self-Assessment Learning Styles Inventory based on the theory propounded by Sternberg [60,65]	Global students have the desire to solve abstract and huge problems [60,65]	RQ4
Local	Factual information	Danish Self-Assessment Learning Styles Inventory based on the theory propounded by Sternberg [60,65]	Local students prefer problems that need detailed and realistic solutions [60,65]	RQ4
Assimilator	Abstract information	Kolb learning style inventory [57]	Assimilators tend to prefer abstract ideas and concepts and are capable of perceiving a diverse range of information [57,66]	RQ4
Task oriented	Factual information	Customized survey designed by Gelbart et al [56]	The task-oriented student pair preferred specific tasks, and they did not always stay involved in all research steps; therefore, they only got a basic idea of what the research was about. They concentrated more on learning the details [56]	RQ4
Research oriented	Abstract information	Customized survey designed by Gelbart et al [56]	Research-oriented students are high achievers who are highly motivated to learn concepts with a deep understanding. They focus on generating abstract ideas and explanations that are connected to theoretical concepts [56]	RQ4

### Information Presentation Preference (RQ1): Multimodal With Higher Tendency Toward Visual Presentation

Of the 8 studies included in this systematic review, 5 (62%) explored the preference of students regarding the type of presentation [41,45,52,54,55]. All these studies reported that students in HDS-related courses prefer visual presentations and benefit more from visualizations than from audio or reading types of presentations. However, all articles also acknowledge that students are multimodal learners and do not have only 1 preference regarding information presentation. In other words, if students prefer visual presentations, such as watching videos, it does not necessarily mean that they do not have any tendency toward reading or other types of presentations; for instance, Micheel et al [41] investigated the learning styles of oncology health care professionals learning precision medicine from web-based educational materials, and their research study showed that 80% of the learners had multimodal learning styles: the majority of the learners (39%) preferred watching, listening, and reading, while the next largest group (19%) preferred watching and reading. Abrahamsson and Dávila Lopez [55] analyzed the learning preferences of graduate students in 5 web-based bioinformatics-related courses and found that 91% of the students preferred synchronous and asynchronous lectures, which include visual presentations, while only 9% favored reading materials. Li and Abdul Rahman [52] analyzed the learning styles of bioinformatics students using the FSILS and found that the majority of the students were visual learners (66%). Holtzclaw et al [45] investigated the learning styles of undergraduate genetics students in a bioinformatics module and reported that the most dominant learning style among the students was visual (82%) compared to verbal (18%). The results from these studies are consistent with other research [41,52,55] highlighting that the majority of students prefer visual presentations. Finally, the study by Diwakar et al [54] also found that HDS students prefer visual presentations. The authors used the Kolb learning style inventory to classify bioinformatics students into multiple learning preferences and found that the majority of learners were classified as assimilators (60%) [54]. Assimilators tend to learn visually and prefer to observe a clear explanation [57]. A summary of the results of the studies is presented in Table 1.

### Collaboration Preference (RQ2): Inconclusive Evidence

Of the 8 included studies, 5 (62%) [45,46,52,53,55] focused on the collaboration preferences of HDS students, and the results were inconclusive (Table 1). Most of these studies (3/5, 60%) [45,52,55] demonstrated that approximately half of the students preferred teamwork, while the other half preferred to work individually. Conversely, 2 (40%) of these 5 studies [46,53] indicated that HDS students had a preference for working in groups.

The study by Holtzclaw et al [45] is 1 (33%) of the 3 studies that show no clear student preference regarding collaboration in HDS. In particular, the authors reported that 54% of the bioinformatics students were found to be active learners, who typically prefer collaborating with peers, and 46% were found to be reflective learners, who have a tendency to work independently [45]. The difference between the 2 groups was not significant enough to conclude that there was a clear preference for collaboration or individual work. Similarly, Li and Abdul Rahman [52] found that more than half (55%) of the undergraduate bioinformatics students in their study were categorized as active learners (a tendency to collaborate with others), with the rest being categorized as reflective learners (a preference to work alone) or neutral. Abrahamsson and Dávila Lopez [55] reported that approximately 50% of the bioinformatics students in their study preferred to work alone on course assignments, while the other half preferred to work in groups (19% preferred to study with the same group for all sessions, 19% preferred to study with different groups, and 12% preferred to work individually in the first sessions and then study in groups).

The study by Sani Ibrahim [46] is 1 (50%) of the 2 studies indicating HDS students’ preference for working in groups. The author reported that 70% of the bioinformatics students participating in the study were active learners who performed better in groups. In addition, the findings from the study by Nielsen and Kreiner [53], who used the Danish Self-Assessment Learning Styles Inventory, demonstrated that students enrolled in an advanced health statistics course had a strong tendency to be external, which shows their preference toward teamwork, with 89.3% of the students scoring as strong or very strong in this dimension (Table 1). This strong preference for external scope style suggests that students are willing to work as a team and communicate with others.

Overall, no consistent conclusion can be drawn based on the studies regarding HDS students’ preference for working individually or in a group. Abrahamsson and Dávila Lopez [55] discuss several possible reasons for this inconsistency: first, the academic level of students may influence their preferences—postgraduate students have a higher research workload and are busier, which may lead to a higher tendency to work alone. Second, the type of assignment can influence students’ working preferences; for example, the authors encouraged students to adopt paired programming for their programming assignments, and this optional approach was adopted by 85% of the bioinformatics students in their study, highlighting the effect of including activities in course design to promote student interactions. Finally, according to the authors, another possible reason could be the course platform because collaboration can be difficult in web-based courses.

### Learning Process Organization Preference (RQ3): Sequential Learning Is More Popular

According to 3 (38%) of the 8 studies [45,52,54], the majority of HDS learners tend to have a sequential learning preference for organizing their learning process. Li and Abdul Rahman [52] found that 62% of their study participants had a sequential learning preference, while Holtzclaw et al [45] reported an even higher percentage of 75% (Table 1). Diwakar et al [54] also supported this conclusion, with 60% of their student population being assimilators, who tend to organize information logically and with clear order [57]. However, we should note that the number of studies that explored this dimension of preference is low, and further research is required to draw strong conclusions.

### Focus Granularity Preference (RQ4): Higher Preference Toward Abstract Information

Of the 8 papers included in this systematic review, 5 (62%) provide evidence regarding the focus of students on abstract versus detailed information [45,46,52-54], with the majority of these papers (4/5, 80%) [46,52-54] agreeing that HDS students prefer main and abstract ideas (refer to Table 1 for further details).

The evidence regarding students’ preferences for detailed or abstract information can be identified from the different learning styles reported (eg, intuitive or sensing, global or local, assimilator, executive, and research or task oriented) in the learning inventories used by these 5 studies. The study by Li and Abdul Rahman [52] found that the percentage of intuitive students (48%) was higher than that of sensing students (approximately 30%), while approximately 20% of the students were neutral in this dimension. Intuitive students prefer to focus on abstract ideas rather than detailed and factual knowledge, and they use a creative approach to problem-solving [58]. Similarly, Sani Ibrahim [46] expanded on the findings of Li and Abdul Rahman [52] and after using their data in addition to Moodle data, concluded that 94% of the bioinformatics students were intuitive. In the study by Diwakar et al [54], students were mostly assimilators (60%), who typically focus on abstract ideas and concepts. In addition, Nielsen and Kreiner [53] showed that HDS students tend to be slightly more global (ie, have the intention to solve abstract problems) rather than local (ie, have the desire to address detailed and realistic problems). Although the difference in the average scores for the 2 groups is small (Table 1), a much higher percentage of students (approximately 30%) scored strongly or very strongly as global compared to local (approximately 11%).

In contrast to the aforementioned studies that indicate a preference for abstract information, Holtzclaw et al [45] found that most students (67%) had a preference for sensing learning, preferring to focus on factual and detailed information.

In addition to the aforementioned 5 studies, Gelbart et al [56] identified 2 learning approaches among high school biology students in a bioinformatics-related course: research oriented (where abstract ideas are valued more highly) and task oriented (where there is attention to detail and focus on factual knowledge). However, this study included only 4 participants (research oriented: 2 and task oriented: 2), with insufficient evidence for addressing the particular RQ.

In conclusion, there is some evidence supporting the inference that HDS students prefer abstract information. However, it should be noted that there are also contradictory findings, and further research is needed to arrive at a more solid conclusion.

## Discussion

### Overview

A total of 8 articles that were published between 2005 and 2021 were included in the synthesis step. The synthesized results show that most HDS learners prefer visual presentations as their learning input. Regarding learning process and organization, they mostly prefer to follow logical, linear, and sequential steps. In addition, they focus more on abstract information, rather than detailed information. In terms of collaboration, HDS students prefer a mix of teamwork and independent work. On the basis of the findings of this systematic review, we provide herein some suggestions for future research and some recommendations for improving the design of HDS courses.

### Recommendations for Course Design

It is known that student preferences can guide course instructors in designing more effective courses [10,22,24]. On the basis of HDS students’ preference for visual presentation of information, it would be beneficial to include more attractive plots, flowcharts, and visual graphics within the course materials to make them more visually impressive.

Given HDS students’ inclination toward sequential learning, where they organize their learning process in logical and clear steps, it would be advantageous to consider a stepwise approach in course design. Including step-by-step instructions for practical implementations or dividing concepts into meaningful sequential parts, may also benefit students; for example, Holtzclaw et al [45] designed a bioinformatics module based on students’ learning styles, containing highly visual components and facilitating sequential learning. On the basis of postcourse feedback, students rated this module as valuable for their educational goals.

In terms of collaboration preferences, there is no consistent conclusion based on existing studies. Therefore, we recommend designing HDS courses in such a way that students can choose freely between individual work and teamwork. This includes coursework where both types of assignments are offered.

Our final suggestion is that, given the evidence regarding the higher focus of HDS students on main and abstract ideas (as opposed to detailed information) and their tendency to apply a creative approach to problem-solving, it would be advantageous to reduce the details in the main course materials and instead include them in an appendix. In addition, creating challenging assignments that prompt reflection on abstract concepts and encourage the use of intuitive approaches for problem-solving can be beneficial for HDS students.

Although the aforementioned recommendations are based on the preferences of the majority of students in the reviewed studies, it is essential for educators to be aware of the heterogeneity of students’ learning preferences and diversify HDS course design accordingly [53]. As the suggestions presented in this systematic review are based on a limited number of available studies, it is essential for educators to carefully consider the context of their specific course and student population when integrating these suggestions into their course design.

### Guidelines for Future Studies

Additional research is needed to explore learning preferences and strategies in HDS courses, especially considering the conflicting findings in certain learning preferences (eg, collaboration preference and preferred focus granularity). In this subsection, we provide some suggestions for future studies.

First, we recommend the use of log data and data mining methods to analyze learning preferences and strategies in HDS courses. The majority of the included studies (6/8, 75%) entirely relied on self-reporting questionnaires or think-aloud protocols [41,53,56]. However, several studies have shown that self-reported inventories may not accurately reflect the actual behavior of learners because the learners may over- or underestimate their learning preferences or learning strategies [10,67]. To avoid this bias, we suggest using log data from learning platforms and data mining methods to accurately analyze the actual behaviors of students and uncover their learning preferences and strategies [10,68,69]. Applying data mining tools on log data can also help to analyze the temporal and dynamic behavior of students over time [70]. Recent studies [10,71] have demonstrated that using data mining tools uncovers students’ preferences or strategies, which are dynamic and highly correlated with their performance [72]. As students may change their learning preferences and strategies throughout their interaction with a course [10,73], it is important to shed light on such changes. In this review, data-driven methods were used only by 2 (25%) of the 8 studies [46,52], which, however, were not well designed because they did not identify students’ learning preferences based on the log data. Instead, they applied the FSILS to identify students’ learning styles and then used the identified learning styles based on self-reported data as labels to train a model using log data; for example, Li and Abdul Rahman [52] only trained a computational model based on self-reported data instead of finding students’ learning preferences using an unsupervised approach.

Second, it is necessary to analyze larger samples to strengthen the results and increase the generalizability of the findings. As mentioned earlier, all included studies except for that by Micheel et al [41] analyzed courses with <100 learners, which can be a limitation depending on the type of analysis conducted. The study by Gelbart et al [56] had a sample size of only 2 pairs of students. Although the study used qualitative analysis, the number of students considered and the information reported about them seem insufficient to support the authors’ conclusion regarding the learning approaches of students [56]. Therefore, researchers, depending on the type of analysis (quantitative or qualitative), should be aware of the importance of having a suitable sample size to minimize the risk of bias in their conclusions [74,75].

Third, most of the included studies (5/8, 62%) did not report the demographic information of students. This is an important omission because students’ nationality, race, and culture may affect their learning preferences [52]. To minimize the impact of other factors on the students’ preferences and capture the preferences related solely to the HDS discipline, future research needs to include a diverse range of learners in terms of nationality, race, and other demographic characteristics. It is worth mentioning that in this systematic review, we examined the learning strategies and preferences of students across different academic levels, but no statistically significant differences were found between the different levels. Nevertheless, it is important to note that students’ academic levels may influence their learning strategies and preferences. This aspect requires further investigation in future studies.

Finally, future studies should focus on students’ learning strategies rather than learning styles because learning strategies are known to provide more useful information about a field in comparison with learning styles [10,13,76]. In addition, previous research has shown that learning strategies are highly associated with students’ academic performance [77,78], while the association between learning styles and performance is controversial [76,79]. Among the 8 included studies, only 1 (12%) [56] discussed the learning strategies of HDS students, which was limited to self-reported data and had a very small sample size. Overall, much more needs to be done to gain comprehensive knowledge about HDS students. We encourage researchers to explore learning strategies in HDS courses using both log data and self-reported data.

### Limitations

A limitation of this systematic review concerns the small number of studies included (n=8). Although we were systematic in our review and synthesis of these 8 studies, we acknowledge that it is a small number of studies, and therefore the results should be interpreted with caution.

Second, the heterogeneity among the included studies required the use of proxies to synthesize the results, and using meta-analysis was impossible due to the diverse measurements used across the studies. Although this systematic review defined meaningful and valid proxies to connect the heterogeneous pieces of evidence in the included studies, the use of different inventories in the studies to measure learning preferences and strategies can affect the accuracy of our findings.

It is worth mentioning that none of the included studies labeled their courses as “health data science” courses; the majority (6/8, 75%) referred to them as bioinformatics courses. It is important to note that in this systematic review, HDS is defined as a discipline in which students use computational methods and tools to analyze biological, health, or medical data. We did not include courses that focus on non–data analytics aspects, such as mobile health or electronic health records. Therefore, the findings of this systematic review may not apply to non–data analytics courses in health informatics.

Regarding the search queries and inclusion criteria, our study only included primary research studies in English published in journal and conference formats. In addition, due to the wide range of terminologies used in the literature to describe learning preferences and strategies, some relevant studies might have been overlooked given the search keywords used in this review; for example, we did not use the keyword “learning approach” in our search query, which could have resulted in additional studies for inclusion.

Moreover, due to the high occurrence of false positives in the search results obtained through SpringerLink and Wiley Online Library, our query for these 2 databases was restricted to studies that included the keyword “student” in their abstracts, which could have led to studies involving health care professionals being overlooked.

Regarding the quality of the included studies, while the MMAT serves as a powerful tool with low bias in assessment [80], it should be acknowledged that the assessment of the quality of included papers can be subjective. However, the 3 reviewers who assessed the quality of the included articles have different academic backgrounds and levels of expertise, which can potentially mitigate the associated bias.

Finally, students’ learning preferences and strategies can be influenced by the mode of course delivery (eg, web based or face-to-face) and course design [11]; therefore, teachers and course designers should not solely rely on the findings of this study without considering other factors that might influence students’ learning strategies and preferences. In addition, some suggestions within this review may specifically apply to web-based courses; for instance, the recommendation to use learning analytics to analyze students’ learning behavior to identify dynamic learning strategies is not feasible for face-to-face courses.

### Conclusions

We reviewed the literature to identify student learning preferences and strategies in HDS courses. The PRISMA guidelines were used, and, as a result, 8 papers were included for narrative synthesis. The synthesis of these studies provided evidence that most HDS students are visual and prefer learning through visual materials, such as videos, diagrams, plots, and so on, as part of their learning process. They also tend to follow logical and sequential steps in their learning process, and they are inclined to focus more on abstract information than on factual and detailed information. Moreover, there is no agreement among existing studies regarding students’ collaboration preferences (teamwork vs independent work). HDS students might prefer to work alone on some assignments, while sometimes they prefer to work as part of a team.

On the basis of the reviewed studies, we recommend including more visual and less detailed materials in HDS courses, accompanied by stepwise instructions.

Furthermore, to address the limitations of existing studies, future research should consider using log data instead of self-reported questionnaires to capture the actual HDS learning experience. Including a large sample of students from different backgrounds and races can also strengthen research results and reduce the impact of other cofactors unrelated to the HDS discipline.

In addition, analyzing the learning strategies of students, rather than their learning preferences, has the potential to yield deep insights into HDS education because learning strategies are more associated with student performance. Overall, because a small number of studies have investigated learning preferences and strategies in HDS courses, further research is needed to draw definitive conclusions.

## Appendix

Multimedia Appendix 1 PRISMA (Preferred Reporting Items for Systematic Reviews and Meta-Analyses) checklists for abstracts and articles.

Multimedia Appendix 2 Study protocol, search queries, excluded and included studies, and characteristics of the included studies.

Multimedia Appendix 3 Summary of the learning inventories used by the included studies.

Multimedia Appendix 4 Mixed Methods Appraisal Tool checklist results, showing the quality of the included studies.

## Abbreviations

FSILS: Felder and Soloman Index of Learning Styles
HDS: health data science
MMAT: Mixed Methods Appraisal Tool
PRISMA: Preferred Reporting Items for Systematic Reviews and Meta-Analyses
RQ: research question

## Data Availability

All data generated or analyzed during this study are included in this published article and its supplementary information files.
