# Supplementary material for: Identifying Learning Preferences and Strategies in Health Data Science Courses: Systematic Review
Source: JMIR Med Educ. 2024 Aug 12;10:e50667. doi: 10.2196/50667 (PMC11347898; doi:10.2196/50667)
Supplement: Multimedia Appendix 2 [file mededu_v10i1e50667_app2.pdf]

Multimedia Appendix 2

Identifying Learning Preferences and Strategies in Health Data Science Courses: Systematic Review

Narjes Rohani[1], Stephen Sowa[2], and Areti Manataki[3]

[1] Usher Institute, University of Edinburgh, Scotland, UK

[2] Moray House School of Education and Sport, University of Edinburgh, Scotland, UK

[3] School of Computer Science, University of St Andrews, Scotland, UK

Narjes.Rohani@ed.ac.uk

stephen.sowa@ed.ac.uk

A.Manataki@st-andrews.ac.uk

Contents

Study protocol.....2

Study Eligibility.....2

Search Methods and Selection of Eligible Studies.....3

Data extraction.....4

Data Synthesis.....5

Presentation of Results.....5

Search queries used for each database.....5

Details of articles excluded at the full-text reviewing stage.....6

Characteristics of the included papers.....17

Study protocol

This protocol, which describes the techniques for the systematic review, was created following the guidelines provided by the Preferred Reporting Items for Systematic Review and Meta-Analysis Protocols (PRISMA-P) checklist [50,38]. The protocol delineates the steps involved in the systematic review, which tries to address the research question regarding the learning strategies or learning preferences of students enrolled in Health Data Science (HDS) – related courses. The conventional PICO format used for structuring questions in a systematic review and the PICO components of the review question are specified in Table 1.

Table 1: The PICO components of the review question

**Component**	**Description**
Population	Learners enrolled in health data science–related courses.
Intervention	Learning preferences or learning strategies.
Comparator	Not applicable (As the focus is on identifying the learning preferences or learning strategies used by students, the comparison component does not apply to this study).
Outcome	Identification of learning preferences or learning strategies used by students in health data science-related courses.

Study Eligibility

All primary research studies which investigated students' learning preferences or learning strategies in HDS–related courses are eligible for inclusion (Table 2). We include both journal and conference articles.

Table 2: Inclusion and exclusion criteria for the systematic review

**Inclusion criteria**	**Exclusion criteria**
The study is primary research (journal article or conference paper) that explores health data science–related (using computational methods to analyse biological, medical, or health-related problems) disciplines.	The study is not about health data science–related courses (for example, we excluded studies about only medical or clinical courses).
The study explored learning strategy, learning approach and preferences of students regarding learning activities in health data science–related courses.	The study is not a conference or journal article. Conference posters and only abstract studies were excluded.
The language of the article is English.	The study is not about learning preference or learning strategy. We excluded articles that are about preferences regarding course delivery type. For example, preference regarding online or face-to-face courses.

We will include any research that has full–text availability and has investigated the learning preference or strategy of students in health data science–related courses. As our target is courses

and learners in health data science–related courses where students use computational tools to analyse medical, biological, or health data, we will exclude studies that have focused on the learning strategy or learning preference of students in clinical or medical–only courses. Our interest lies in understanding the learning strategy or learning preference of students in interdisciplinary courses related to HDS, such as bioinformatics, biostatistics, health data science, biomedical sciences, and courses that utilize computational methods for the analysis of medical or biological data.

Search Methods and Selection of Eligible Studies

The search strategy consists of two primary components: a learning component that will be utilized to identify research works related to learning strategy or learning preference and a discipline-related component that aims to identify research works related to HDS.

Table 3: Keywords for searching the literature.

Learning component	Discipline component
("learning strategies" OR "learning tactics" OR "learning practices" OR "learning styles" OR "learning preference")	(biostatistics OR "computational biology" OR bioinformatics OR "biomedical science" OR neuroinformatics OR "health data analysis" OR pharmacogenomics OR "precision medicine" OR "health informatics")

The following databases will be searched independently: PubMed, ACM Digital Library, Web of Science, Cochrane Library, Wiley Online Library, ScienceDirect, Springer Link, EBSCOhost, ERIC, and IEEE Xplore. We supplement the literature search by employing Google Scholar (only results of the 10 first pages will be screened) to find potentially missed articles. The search strings will be appropriately adapted for each database. We will include only published articles in English.

To ensure that the references are up to date if more than 6 months elapse between the initial search and the completion of data extraction and analysis, an additional search will be performed. If conducted, the second search will follow the same methodology employed in the initial search. By conducting this search within the original databases, it is expected to capture both the original studies and any new studies published since the previous search. The search results will be compared to ensure that all the original citations are included. Search result citations for literature will be loaded into and managed in EndNote 20.2.0 software and de-duplicated automatically.

The literature screening process will be conducted in two stages, independently by two reviewers. Firstly, a screening of titles and abstracts will be performed on all papers retrieved. Subsequently, both reviewers will conduct a full-text review of the included papers following the abstract screening. All questions will be answered using "yes," "no," or "unsure" responses. In the event of any discrepancies or disagreements in the included list between the two reviewers, a third screener will be engaged to resolve conflicts.

Table 5: Screening questions to be used for the inclusion of articles.

Screening question	Yes - Include	No - Exclude	Unsure - Include
Does the study investigate the learning strategies or	Yes – the study explored the learning strategies or	No – the study is not about learning strategy or preference.	Unsure – it is not clear whether the study reported the learning strategy

preferences of students?	preferences of students.		or preference of students.
Does the study explore health data science-related students?	Yes – the study analysed students in health data science-related courses such as bioinformatics, precision medicine, etc.	No – the study is not about health data science-related courses and students. For example, it is about clinical courses without any computational aspect in the course.	Unsure – it is not clear whether the study participants are in a health data science course or not.
Is the study a published conference or journal article?	Yes	No	Not applicable

Data extraction

Both reviewers will utilise a standardised Microsoft Word form for extracting and documenting data. The data they extract will include the following categories:

– **Publication characteristics**: This includes details such as the publication title, journal of publication, authors of the publication, and publication year.

– **Methodological features**: various methodological aspects, such as the participants' field and course name, the number of participants, the method of analysis employed, the type of input data used, the delivery type of the course, students' degree level, the study subject, and any inventory utilised.

– **Learning preference or strategy**: information regarding reported learning preferences or strategies, along with the corresponding percentage of students exhibiting each learning preference or strategy.

After the initial extraction, both reviewers will cross-check the extracted data to ensure accuracy. Any discrepancies or inconsistencies will be resolved through discussion and consensus between the two reviewers. Subsequently, both reviewers independently will recheck the extracted data and address any additional discrepancies through further discussion.

– **Study quality assessment:** The quality of the articles included will be assessed by both of the screeners using the Mixed Methods Appraisal Tool (MMAT) [49]. The MMAT Excel file containing the criteria for assessing study quality will be filled out by the screeners.

Data Synthesis

We will use narrative synthesis [48], given the fact that this synthesis method is more appropriate for our research questions and education field.

Presentation of Results

The search strategy employed for each database will be documented. To visually represent the screening process and reasons for study exclusion, a flow chart adhering to the PRISMA guidelines [51] will be utilised. Additionally, a summary table will be generated to present the results, highlighting the key information from the papers included.

Search queries used for each database

Table 4: Queries used for each database for finding articles

Database	*Query*
ACM Digital Library	[[Full Text: "learning strategies"] OR [Full Text: "learning tactics"] OR [Full Text: "learning practices"]] AND [[Full Text: biostatistics] OR [Full Text: "computational biology"] OR [Full Text: bioinformatics] OR [Full Text: "biomedical science"] OR [Full Text: neuroinformatics] OR [Full Text: "health data analysis"] OR [Full Text: pharmacogenomics] OR [Full Text: "precision medicine"]] AND [Full Text: student] AND [Full Text: education]
Web of Science	("learning strategies" OR "learning tactics" OR "learning practices" OR "learning styles" OR "learning preference")AND (biostatistics OR "computational biology" OR bioinformatics OR "biomedical science" OR neuroinformatics OR "health data analysis" OR pharmacogenomics OR "precision medicine" OR "health informatics") AND student AND education
Cochrane Library	("learning strategies" OR "learning tactics" OR "learning practices" OR "learning styles" OR "learning preference")AND (biostatistics OR "computational biology" OR bioinformatics OR "biomedical science" OR neuroinformatics OR "health data analysis" OR pharmacogenomics OR "precision medicine" OR "health informatics") AND student AND education in All Text - (Word variations have been searched)
Wiley Online Library	("learning strategies" OR "learning tactics" OR "learning practices" OR "learning styles" OR "learning preference")AND (biostatistics OR "computational biology" OR bioinformatics OR "biomedical science" OR neuroinformatics OR "health data analysis" OR pharmacogenomics OR "precision medicine" OR "health informatics") AND student AND education" in Abstract
PubMed	("learning strategies" OR "learning tactics" OR "learning practices" OR "learning styles" OR "learning preference")AND (biostatistics OR "computational biology" OR bioinformatics OR "biomedical science" OR neuroinformatics OR "health data analysis" OR pharmacogenomics OR "precision medicine" OR "health informatics") AND student AND education"

IEEE Xplore	((("Abstract":"Learning strategy") OR ("Abstract":"Learning tactic") OR ("Abstract":"learning style") OR ("Abstract":"learning preference") OR ("Abstract":"learning practice")) AND (("Full Text Only":"precision medicine") OR ("Full Text Only":"bioinformatics")OR ("Full Text Only":"health informatics") OR ("Full Text Only": biostatistics) OR ("Full Text Only": neuroinformatics) OR ("Full Text Only": "biomedical science")OR ("Full Text Only": "health data analysis") OR ("Full Text Only": pharmacogenomics)OR ("Full Text Only": "computational biology")) AND (("Full Text Only": education)OR("Full Text Only": student)))
ScienceDirect	Find articles with these terms.<br>(biostatistics OR bioinformatics OR "biomedical science" OR "computational biology" OR "health informatics" OR "precision medicine" OR neuroinformatics OR "health data analysis" OR pharmacogenomics)<br>Title, abstract or author-specified keywords<br>("learning strategy" OR "learning tactic" OR "learning practice" OR "learning style" OR "learning preference") AND student
Springer Link	(("learning strategies" OR "learning tactics" OR "learning practices" OR "learning styles" OR "learning preference")AND (biostatistics OR "computational biology" OR bioinformatics OR "biomedical science" OR neuroinformatics OR "health data analysis" OR pharmacogenomics OR "precision medicine" OR "health informatics") AND student AND education) in abstract
EBSCOhost	("learning strategies" OR "learning tactics" OR "learning practices" OR "learning styles" OR "learning preference")AND (biostatistics OR "computational biology" OR bioinformatics OR "biomedical science" OR neuroinformatics OR "health data analysis" OR pharmacogenomics OR "precision medicine" OR "health informatics") AND student AND education
ERIC	("learning strategies" OR "learning tactics" OR "learning practices" OR "learning styles" OR "learning preference")AND (biostatistics OR "computational biology" OR bioinformatics OR "biomedical science" OR neuroinformatics OR "health data analysis" OR pharmacogenomics OR "precision medicine" OR "health informatics") AND student AND education

Google Scholar, using the same keywords as used for other databases, was used for manual searching. Because Google Scholar finds numerous false positive results and it is not practical to check all resulting pages, we limited the screening to only the initial 10 pages.

Details of articles excluded at the full-text reviewing stage

Table 4: Excluded articles during full-text screening

Study title	Authors	Year	Include	Reason

Using multiple self-regulated learning measures to understand medical students' biomedical science learning	Gandomkar, Roghayeh Yazdani, Kamran Fata, Ladan Mehrdad, Ramin Mirzazadeh, Azim Jalili, Mohammad Sandars, John	2020	No	Other, Not enough information or evidence that the course is HDS-related. It seems the course/task does not have any computational aspect.
Self-regulated learning processes of medical students during an academic learning task	Gandomkar, Roghayeh Mirzazadeh, Azim Jalili, Mohammad Yazdani, Kamran Fata, Ladan Sandars, John	2016	No	Other, Not enough information or evidence that the course is HDS-related. It seems the course or task does not have any computational aspect.
Language Focus for Genetics and Molecular Biology Students	Lidbury, B. A. Informat Resources Management, Assoc	2013	No	Out of scope
Component-Based Approach for Educating Students in Bioinformatics	Poe, D. Venkatraman, N. Hansen, C. Singh, G.	2009	No	Not learning strategy or preference

Personality, Preferences, Satisfaction, and Achievement in a Biostatistics Course: Traditional versus Flipped Classrooms in Nursing Education	Yanez, A. M.<br>Adrover-Roig, D.<br>Bennasar-Veny, M.	2023	No	Not HDS course or student
Youubi: Open software for ubiquitous learning	de Sousa Monteiro, Bruno Gomes, Alex Sandro, Mendes Neto, Francisco Milton	2016	No	Out of scope
Workload, Study Methods, and Motivation of Students within a BVSc Program	Tim J. Parkinson, Marg Gilling, Gordon T. Suddaby	2006	No	Not HDS course or student
WHAT WE CAN LEARN FROM OUR STUDENTS? USE OF STUDENT REFLECTIONS AS TOOLS TO IMPROVE TEACHING STRATEGIES	Sullivan, S. O.<br>McGlynn, H.	2010	No	No full-text access
Web-based learning in undergraduate medical education: development and assessment of an online course on experimental surgery	Bernardo, Viviane<br>Ramos, Monica Parente<br>Plapler, Helio<br>de Figueiredo, Luiz Francisco Poli<br>Nader, Helena B.<br>Anção, Meide Silva<br>von Dietrich, Carl P.<br>Sigulem, Daniel	2004	No	Not HI course or student

Understanding the massive open online course (MOOC) student experience: An examination of attitudes, motivations, and barriers	Shapiro, Heather B.<br>Lee, Clara H.<br>Wyman Roth, Noelle E.<br>Li, Kun<br>Çetinkaya-Rundel, Mine<br>Canelas, Dorian A.	2017	No	Not HDS course or student
Undergraduate quantitative biology impact on biology preservice teachers	Mayes, Robert<br>Long, Tammy<br>Huffling, Lacey<br>Reedy, Aaron<br>Williamson, Brad	2020	No	Not HDS course or student
Trajectories of learning approaches during a full medical curriculum: impact on clinical learning outcomes	Giovanni PiumattiSissel GuttormsenBarbara ZurbuchenMilena AbbiatiMargaret W. GerbaseAnne Baroffio	2021	No	Not HDS course or student
Self-directed student research through analysis of microarray datasets: A computer-based functional genomics practical class for masters-level students	Grenville-Briggs, Laura J.<br>Stansfield, Ian	2011	No	Not learning strategy or preference

The role of critical thinking skills and learning styles of university students in their academic performance	Ghazivakili, Zohre<br>Nia, Roohangiz<br>Norouzi<br>Panahi, Faride<br>Karimi, Mehrdad<br>Gholsorkhi, Hayede<br>Ahmadi, Zarrin	2014	No	Not HDS course or student
Research Report: Learning Styles of Biomedical Engineering Students	Dee, Kay C.<br>Nauman, Eric A.<br>Livesay, Glen A.<br>Rice, Janet	2002	No	Not HDS course or student
The Relationship Between Learning and Study Strategies Inventory (LASSI) and Academic Performance in Medical Schools	Khalil, Mohammed K.<br>Hawkins, H. Gregory<br>Crespo, Lynn M.<br>Buggy, James	2017	No	Other
Profile of Students' Metacognitive Skill Based on Their Learning Style	Palennari, Muhiddin<br>Taiyeb, Mushawwir<br>Saenab, Siti	2018	No	Not HDS course or student
Prediction of academic achievement based on learning strategies and outcome expectations among medical students	Sakineh NabizadehSepideh HajianZohre SheikhanFatemeh Rafiei	2019	No	Not HDS course or student

Motivational profiles of medical students: Association with study effort, academic performance and exhaustion	Charles E. CunninghamKen DealAlan NevilleHeather RimasLynne Lohfeld	2006	No	Not HDS course or student, Duplicate
Making the Learning in Medical Field More Attractive by Using Multimedia and Videos Tools: A Case Study	Dascalu, C. G.<br>Antohe, M. E.<br>Zegan, G.<br>Dimitriu, G.	2018	No	Not learning strategy or preference
Leveraging an Innovative Teaching Strategy to Identify Use of Biostatistics by Advanced Practice Nurses	Gillespie, Gordon L.<br>Hopgood, Daniel	2021	No	Not learning strategy or preference, Duplicate
Introduction to the Symposium “Leading Students and Faculty to Quantitative Biology through Active Learning”	Waldrop, Lindsay D.<br>Miller, Laura A.	2015	No	Not learning strategy or preference
An interactive, multi-modal Anatomy workshop improves academic performance in the health sciences: a cohort study	Leslie L. NicholsonDarren ReedCliffton Chan	2016	No	other
Integrating a Primer Course in Biostatistics into the Haematology Practicals of First-Year Medical Students in India	Vaz, Mario	2001	No	Not learning strategy or preference
Innovative strategies in teaching of biomedical sciences to health professionals	Niger J Physiol Sci	2005	No	other

Increasing student engagement with course content in graduate public health education: A pilot randomized trial of behavioral nudges	Samantha GarbersAllyson D. CrinklawAdam S. BrownRoxanne Russell	2023	No	Not HDS course or student
Incorporating wiki technology in a traditional biostatistics course: Effects on university students' collaborative learning, approaches to learning and course performance	Fong, SM<br>Chu, Samuel Kai Wah<br>Lau, Wilfred WF<br>Doherty, Iain<br>Hew, Khe Foon Timothy	2017	No	No HDS course or student
Improving teaching in different disciplines of natural science and mathematics with innovative technologies	Umitzhan KossybayevaBagit ShaldykovaDanna AkhmanovaSvetlan a Kulanina	2022	No	Not learning strategy or preference
How to Encourage a Lifelong Learner? The Complex Relation between Learning Strategies and Assessment in a Medical Curriculum	van Woezik Tamara,<br>Koksma Jur,<br>Reuzel Rob,<br>Jaarsma Debbie,<br>Jan van der Wilt Gert,	2020	No	Other, mixed students in medical and biomedical science fields and no enough information about the task and the course
Genome annotation in a community college cell biology lab	Beagley, C. T.	2013	No	Not learning strategy or preference

From biology to mathematical models and back: teaching modeling to biology students, and biology to math and engineering students	Chiel, Hillel J<br>McManus, Jeffrey M<br>Shaw, Kendrick M	2010	No	Not learning strategy or preference
Fostering Effective Learning Strategies in Higher Education – A Mixed-Methods Study	Biwer, Felicitas<br>Egbrink, Mirjam G. A. oude<br>Aalten, Pauline<br>de Bruin, Anique B. H.	2020	No	Other, mixed students in medical and biomedical science fields and no enough information about the task and the course
First year medical students' learning style preferences and their correlation with performance in different subjects within the medical course	Hernández-Torrano, Daniel<br>Ali, Syed<br>Chan, Chee-Kai	2017	No	Not HDS course or student
Factors Affecting Postgraduate Dental Students' Performance in a Biostatistics and Research Design Course	El Tantawi, M. M. A.	2009	No	Not HDS course or student
Exploration of a Collaborative Self-Directed Learning Model in Medical Education	Kemp, Kyeorda<br>Baxa, Dwayne<br>Cortes, Claudio	2022	No	Not HDS course or student
Evolutionary Medicine and the Medical School Curriculum: Meeting Students Along Their Paths to Medical School	Jay B. Labov	2011	No	Not learning strategy or preference

Differences in students' perceptions of learning compulsory foundation biochemistry in the health sciences professions	Minasian-Batmanian, L. C.<br>Lingard, J.<br>Prosser, M.	2005	No	No full-text access
Development of a Secondary Dental-Specific Database for Active Learning of Genetics in Dentistry Programs	Sharmin, N.<br>Chow, A. K.<br>Govia, S.	2022	No	Out of scope
Developing and Implementing Cloud-Based Tutorials That Combine Bioinformatics Software, Interactive Coding, and Visualization Exercises for Distance Learning on Structural Bioinformatics	Engelberger, Felipe<br>Galaz-Davison, Pablo<br>Bravo, Graciela<br>Rivera, Maira<br>Ramirez-Sarmiento, Cesar A.	2021	No	Not learning strategy or preference
Data Science in Undergraduate Medicine: Course Overview and Student Perspectives	Doudesis, Dimitrios<br>Manataki, Areti	2021	No	Not learning strategy or preference

Conceptions of learning factors in postgraduate health sciences master students: a comparative study with non-health science students and between genders	Campos, Fernando<br>Sola, Miguel<br>Santisteban-Espejo, Antonio<br>Ruyffelaert, Ariane<br>Campos-Sánchez, Antonio<br>Garzón, Ingrid<br>Carriel, Víctor<br>de Dios Luna-Del-Castillo, Juan<br>Martin-Piedra, Miguel Ángel<br>Alaminos, Miguel	2018	No	Not HDS course or student
A combination of active learning strategies improves student academic outcomes in first-year paramedic bioscience	Sinnayah, P.<br>Rathner, J. A.<br>Loton, D.<br>Klein, R.<br>Hartley, P.	2019	No	Not HDS course or student
Challenge-based instruction in biomedical engineering: A scalable method to increase the efficiency and effectiveness of teaching and learning in biomedical engineering	Harris, Thomas R.<br>Brophy, Sean P.	2005	No	Not learning strategy or preference
Building Healthcare Professionals in a Medical Science Curriculum		2011	No	Not HDS course or student

Bioinformatics-Based Activities in High School: Fostering Students' Literacy, Interest, and Attitudes on Gene Regulation, Genomics, and Evolution	Martins, A.<br>Fonseca, M. J.<br>Lemos, M.<br>Lencastre, L.<br>Tavares, F.	2020	No	Not learning strategy or preference
Attitudes to concept maps as a teaching/learning activity in undergraduate health professional education: influence of preferred learning style	Laight, D. W.	2004	No	Not HDS course or student
Assessment of a block curriculum design on medical postgraduates' perception towards biostatistics: a cohort study	Li, Chen<br>Wang, Ling<br>Zhang, Yuhai<br>Li, Chanjuan<br>Xu, Yongyong<br>Shang, Lei<br>Xia, Jielai	2018	No	Not learning strategy or preference
"On the job" learning: A bioinformatics course incorporating undergraduates in actual research projects and manuscript submissions	Smith, Jason T.<br>Harris, Justine C.<br>Lopez, Oscar J.<br>Valverde, Laura<br>Borchert, Glen M.	2015	No	Not learning strategy or preference

Characteristics of the included papers

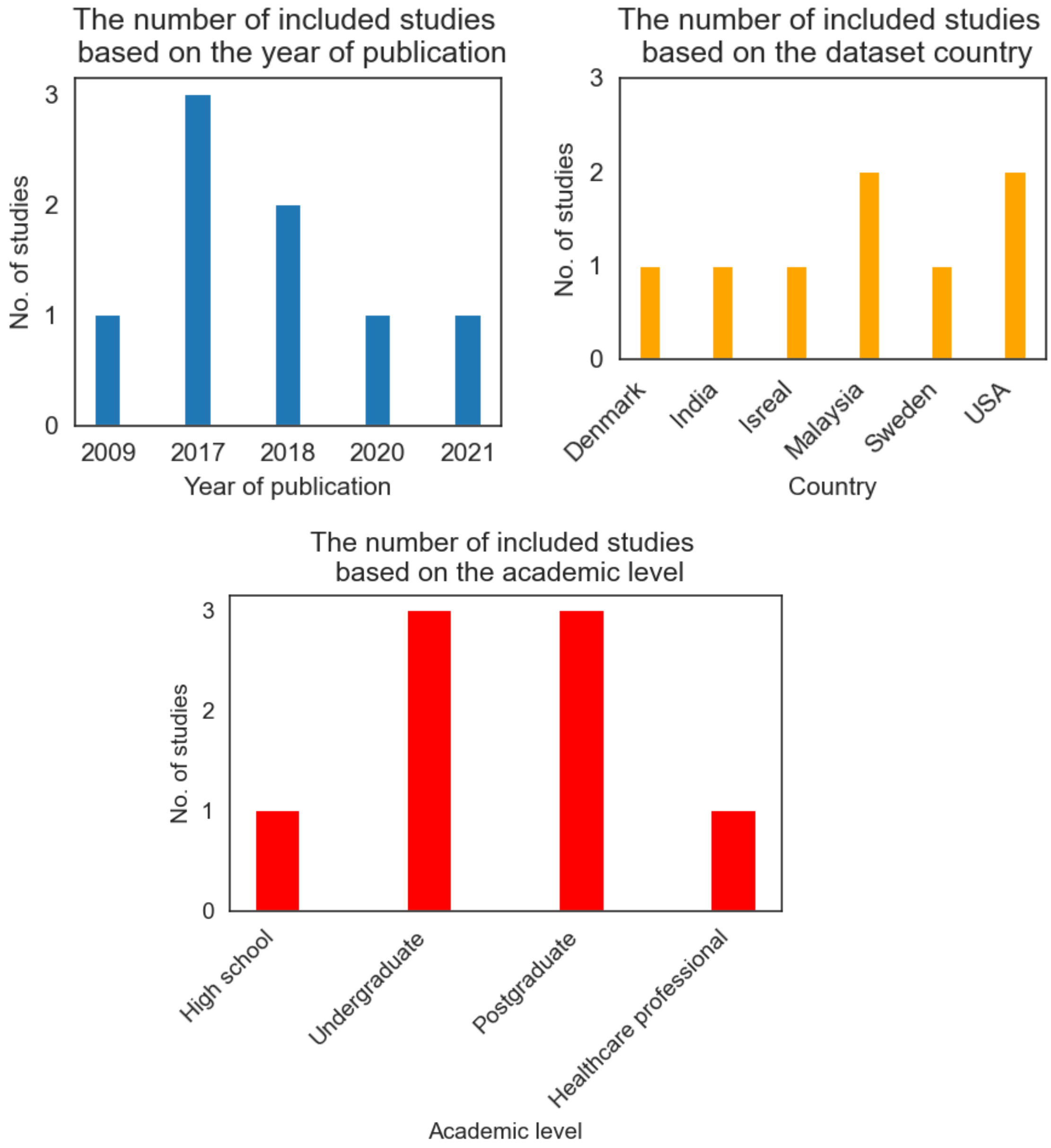

Figure 1: Characteristics of included papers: The subplots display the number of studies included in the systematic review categorised by year of publication, country, and academic level of the learners, respectively.

Multimedia Appendix 2

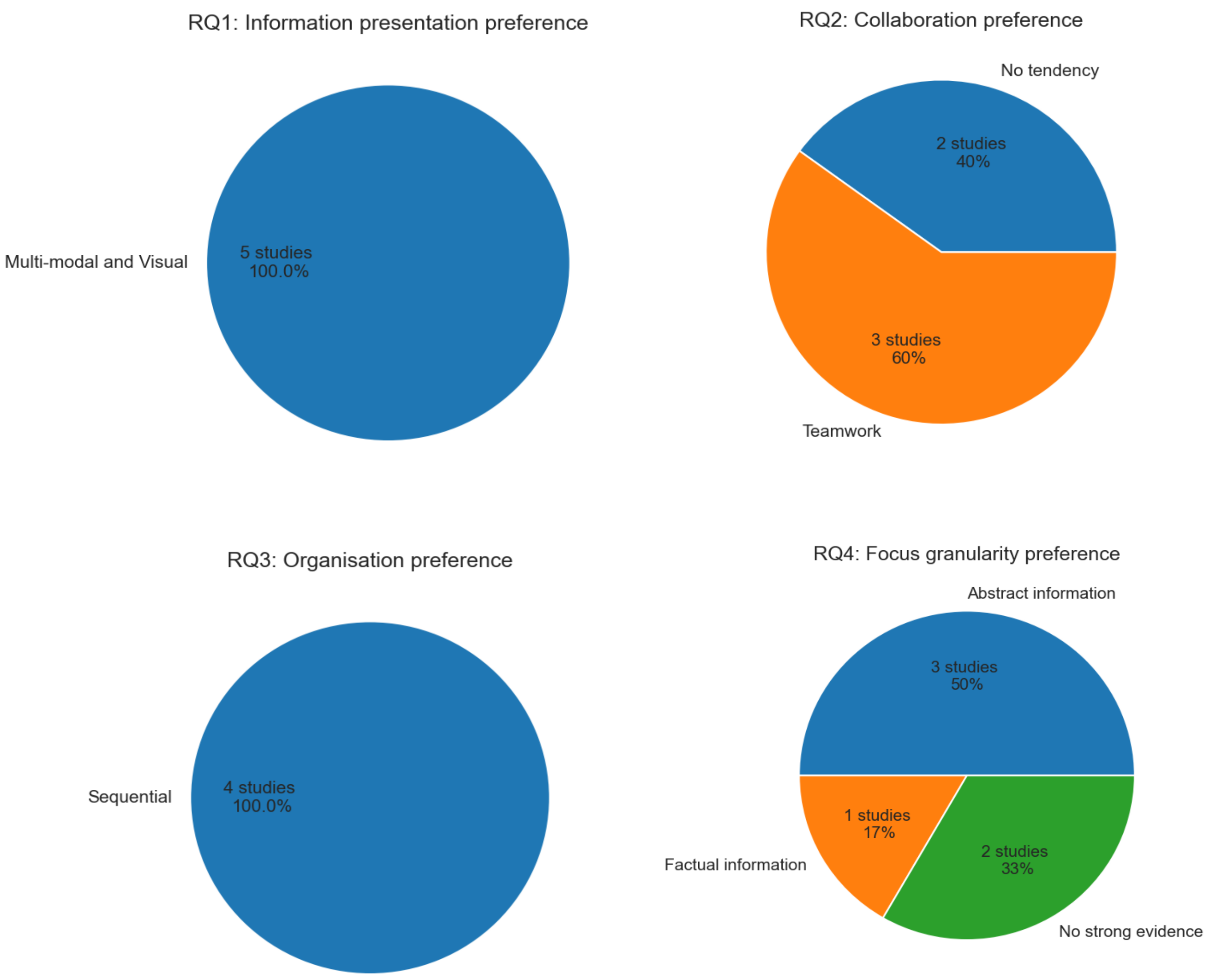

Figure 2: The number of studies supporting each learning preference. The no strong evidence show the number of studies that reported a higher value to preference toward abstract information compared to factual information, but the percentage is not strong enough.
