# Supplementary material for: Identifying Learning Preferences and Strategies in Health Data Science Courses: Systematic Review
Source: JMIR Med Educ. 2024 Aug 12;10:e50667. doi: 10.2196/50667 (PMC11347898; doi:10.2196/50667)
Supplement: Multimedia Appendix 3 [file mededu_v10i1e50667_app3.pdf]

Multimedia Appendix 3

Identifying Learning Preferences and Strategies in Health Data Science Courses: Systematic Review

Narjes Rohani[1], Stephen Sowa[2], and Areti Manataki[3]

[1] Usher Institute, University of Edinburgh, Scotland, UK

[2] Moray House School of Education and Sport, University of Edinburgh, Scotland, UK
[3] School of Computer Science, University of St Andrews, Scotland, UK
Narjes.Rohani@ed.ac.uk
stephen.sowa@ed.ac.uk
A.Manataki@st-andrews.ac.uk

1 Background Information: Learning Inventories

In this appendix, we provide some background information on learning inventories [58,61,63], which are questionnaires that are typically used for discovering learning preferences and strategies. The inventories categorise students into different groups based on various learning dimensions, such as student motivation, the preferred way for information presentation (such as visual or auditory) and their approach to assimilating concepts [59]. Four inventories were employed by studies included in this systematic review, and we discuss them here to help readers follow the Result section.

1. **Felder & Soloman Index Learning Survey (FSILS):** This inventory was designed by Richard Felder and Barbara Soloman in 1991 [58,59]. It measures the learning style of students across four dimensions:
   - **Active or Reflective:** This dimension indicates how students like to process information. Active learners prefer working actively with learning materials to learn, while reflective learners process information by thinking rather than participating in practical activities. Also, active students tend to work as a group and discuss learning materials with others; while reflective learners are inclined to work alone or communicate with a close friend instead of a large group.
   - **Sensing or Intuitive:** This dimension shows how students perceive knowledge. Sensing learners are interested in facts and concrete concepts. They prefer exploring detailed information and intend to solve problems with standard approaches rather than innovative ones. Moreover, they are realistic and hence, prefer practical information that can be used in the real world. Conversely, intuitive learners are enthusiastic about abstract information, such as theories and the deep meaning of learning materials. In comparison to the sensing group, this group are more innovative/creative and can discover possible relationships between the concepts.
   - **Visual or Verbal:** This dimension indicates the preference of students about the medium of information presentation. Visual learners prefer to observe information in pictures, diagrams, flowcharts, and other types of visual resources, whereas verbal learners learn better through textual representations (both written and spoken).
   - **Sequential or Global:** This dimension shows how learners like to organise their understanding process. Sequential students have a linear learning process, which means they prefer to gain knowledge by following incremental and logical steps. On the other hand, global learners have holistic and systematic thinking and prefer large leaps for learning. They interact with learning materials randomly without following logical connections, but surprisingly after gaining enough knowledge, they comprehend the whole picture. They are capable of solving sophisticated assignments by using innovative approaches and connecting different concepts.

However, they cannot explain how they find the solution due to their focus on the whole picture rather than the details.

2. **Danish Self-Assessment Learning Styles Inventory (D-SA-LSI) based on Sternberg's theory:** This inventory is based on the Sternberg and Wagner thinking style designed in 1997 [60] to measure five dimensions of mental self-regulation: function, form, level, scope, and learning. DSA-LSI classifies students into 14 learning styles embedded in the five categories [65]:
   - **Functions:**
     - **Legislative:** Students in this learning style have an intention for strategy creation, goal setting, and problem design, as well as working independently. They are inclined to accomplish tasks alone.
     - **Executive:** They tend to discover clear-cut approaches for solving problems, and they employ predefined actions and rules.
     - **Judicial:** They have the desire to evaluate and criticise the methods, ideas, and results.
   - **Forms:**
     - **Monarchic:** They prefer to complete a single task or goal, which is the most vital one, at that moment.
     - **Hierarchic:** They prefer to have a systematic and balanced approach to address a hierarchy of goals with different levels of importance. In other words, they seek to solve a prioritised problem first.
     - **Oligarchic:** They are multi-task learners, willing to fulfil multiple goals with an equal level of importance simultaneously.
     - **Anarchic:** They randomly select goals to achieve, without considering their importance. They do not like the systematic approach to solving an assignment.
     - **Democratic:** They seek to find the most widely accepted solution for a problem because they do not rely only on their goal and attitudes but rather consider others' opinions too.
   - **Levels:**
     - **Global:** They have the desire to solve abstract and huge problems.
     - **Local:** They prefer problems that need detailed and realistic solutions.
   - **Scopes:**
     - **Internal:** They prefer to work alone without communication with others. Therefore, they would like to apply their idea all by themselves.
     - **External:** They tend to work in a team and collaborate with others to solve problems.
   - **Learning:**
     - **Conversative:** They prefer well-known settings to avoid uncomfortable and complex situations.
     - **Progressive:** They seek unknown and challenging situations, and they look for maximising changes.

3. **Kolb learning style inventory:** This tool was published by David Kolb in 1984 [62] and it categorises the learners into four groups based on their internal cognitive processes:

   - **Divergent:** Students with this learning style tend to gather information to look at concrete problems from various aspects. They prefer to observe rather than perform. Also, they effectively benefit from their imagination for idea generation and brainstorming. They enjoy working as a group to exchange feedback.

- **Assimilator:** Assimilators tend to abstract ideas and concepts and are capable of perceiving a diverse range of information. They can also organise the gained knowledge in a logical and clear format. Theories are more important for them than practical aspects of knowledge. In an online learning environment, assimilators are interested in watching videos, and figures, and gaining knowledge about the facts. Moreover, they prefer to have time to contemplate the materials.
- **Converger:** Students with this learning style are engrossed in putting theories and gained knowledge into practice to solve real-world problems. They also prefer to work on technical assignments.
- **Accommodator:** Accommodator learners are more intuitive rather than logical, and they prefer to rely on others' findings and employ them in their practical and experimental analyses.
